# Innovative stain-free technique for high-resolution imaging of virus particles via standard transmission electron microscopy

**DOI:** 10.1016/j.heliyon.2024.e26172

**Published:** 2024-02-13

**Authors:** Raja muthuramalingam Thangavelu, Washington Luis da Silva

**Affiliations:** Department of Plant Pathology and Ecology, The Connecticut Agricultural Experiment Station, New Haven, CT, USA

**Keywords:** Virus structures, Crystallization, Transmission electron microscopy (TEM), Stain-free, High-resolution imaging

## Abstract

This research presents a groundbreaking approach in virus-related research, addressing challenges in electron microscopy (EM). This imaging technique has been crucial in exploring virus structures; however, traditional methods involve complex sample preparations and the risk of contamination. Herein, we introduce an approach that overcomes these obstacles, enabling high-resolution virus imaging without toxic staining procedures. Focusing on Begomovirus particles, an economically significant plant virus genus, our images confirm their non-enveloped structure and their twin icosahedral symmetry. Our methods involve sample collection, purification, and crystallization, followed by transmission electron microscopy - selected area electron diffraction (TEM-SAED) analysis. Notably, this study achieves 2D and 3D virus imaging through standard TEM, providing a new avenue for virus structure analysis and advancing virus-related research. Remarkable high image quality stemmed from the crystallization process, offering exciting possibilities for improving virus research and diagnosis while eliminating staining limitations.

## 1 Introduction

Electron microscopy (EM) is an indispensable tool in advancing virus-related research, greatly contributing to the exploration, diagnosis, and understanding of various viruses and their intricate interactions with host organisms [1,2]. Traditionally, the gold standard for examining the ultrafine structures of viruses has been cryo-transmission electron microscopy (cryo-TEM) combined with negative staining (NS). However, achieving pristine virus images through EM remains a complex endeavor, requiring meticulous physical and chemical preparations before imaging can occur [3]. Nevertheless, this pursuit is not without its challenges. The specter of cross-contamination during sample preparation often impedes the acquisition of high-quality virus images, and the use of negative staining can introduce ambiguity in identifying virus particles accurately [4,5]. Consequently, the need for innovative techniques becomes apparent as we strive to overcome these obstacles and improve virus structure visualization.

This communication introduces a simple yet groundbreaking method that addresses the challenges associated with conventional EM techniques. It enables the acquisition of high-resolution virus images without the need for potentially hazardous negative-staining procedures, such as those involving chemically toxic and radioactive substances like Uranyl Acetate [6]. The study focuses on Begomovirus particles, economically significant plant viruses within the Geminiviridae family, isolated from squash leaf samples [7]. These viruses possess a non-enveloped structure with single-stranded circular DNA and exhibit twin icosahedral symmetry (T = 1), measuring on average 38 nm in length and 20–28 nm in diameter. Their capsids comprise of 22 pentameric capsomers, each composed of 110 capsid proteins. The structure of Geminivirus has been extensively studied using cryo-TEM due to its significant economic importance in agriculture [[8], [9], [10]]. Geminiviruses are plant-infecting and insect-transmissible, posing a significant threat to many staple crops worldwide such as cassava, beans, and cucurbits, leading to reduced agricultural productivity and economic losses. The impact of Geminiviruses on agriculture is substantial, with estimated yield losses amounting to billions of dollars annually [11,12]. This innovative approach provides fresh insights into general virus structural properties and underscores the potential of stain-free TEM to advance virus research. By eliminating the limitations of staining procedures, this method offers exciting possibilities for enhancing our understanding of virus structures and improving virus-related research and diagnosis.

## 2 Methods and findings

Leaf samples from squash plants displaying symptoms of Begomovirus infection were collected from the field, and the infection status of the samples was confirmed through PCR of *Squash Leaf Curl China Virus* (SLCCNV), as reported earlier [7]. The virus particles were then purified using the Cesium sulfate (Cs_2_SO_4_) density gradient ultracentrifugation (CP100WX, HITACHI, JAPAN) [13]. The fractionated virus proteins were carefully collected and dialyzed against 0.1 M phosphate buffer to remove any residual Cesium sulfate. The sample was further purified using gel filtration chromatography. To assess the heterogeneity of the purified virus particles in solution, gel electrophoresis was conducted, followed by Western blot analysis using antiserum provided by Dr. V.G. Malathi, Principal Scientist and Virologist at the Indian Agricultural Research Institute, India.

### 2.1 Crystallization of virus particles

Crystallizing a protein without a well-established protocol is extremely challenging. Successful crystallization relies on factors such as pH (close to the Isoelectric point), temperature, solvents nature, precipitants composition, and the presence of added ions or ligands. Crystals form when protein molecules precipitate from a supersaturated solution. In this research, our primary objective was to examine the structural properties of purified virus samples using single-crystal X-ray diffraction. This study focused on a unique aspect: analyzing a single virus particle that had never been crystallized before and had never been subjected to X-ray diffraction analysis. The crystallization trials were conducted using the standard Hampton research kits, employing the Hanging drop crystallization method at 18 °C [14]. This technique utilizes diffusion to establish equilibrium and requires only a small amount of protein. Specifically, a drop of 1 μL of purified protein solution was mixed with an equal volume of precipitant solution inside an inverted siliconized microscope glass coverslip. The coverslip was then placed upside down over a small well containing 0.5–1 ml of the precipitating solution. To ensure proper sealing of the chamber, grease (Dow Corning Vacuum Grease) was applied to the circumference of the well before placing the coverslip. The precipitant was allowed to equilibrate against a 1:1 ratio of protein and precipitant, resulting in the appearance of a well-shaped crystal after a month. The composition of this successful crystal comprised 0.5 M Ammonium sulfate, 0.1 M Sodium citrate tribasic dihydrate at pH 5.6, and 1.0 M Lithium sulfate monohydrate.

We monitored the crystal setup and the growth of a single crystal daily under a compound light microscope. After three weeks, the crystals were produced. The single crystal was mounted, and X-ray diffraction (XRD) data were recorded, although the single crystal was produced, there was no diffraction. Thus, we sought an alternative approach. We opted to investigate the crystallized virus samples using TEM-SAED (Selected Area Electron Diffraction). This imaging technique involves the same sample preparation as TEM but without the need for a negative staining procedure. Therefore, we initially examined the samples using TEM and subsequently with SAED. While conducting TEM visualization, we uncovered a remarkable discovery, samples could be observed and pictured without staining. Consequently, we decided to expand the TEM imaging procedure before proceeding with the SAED analysis.

TEM Imaging of Virus Particles.

**Fig. 1:**
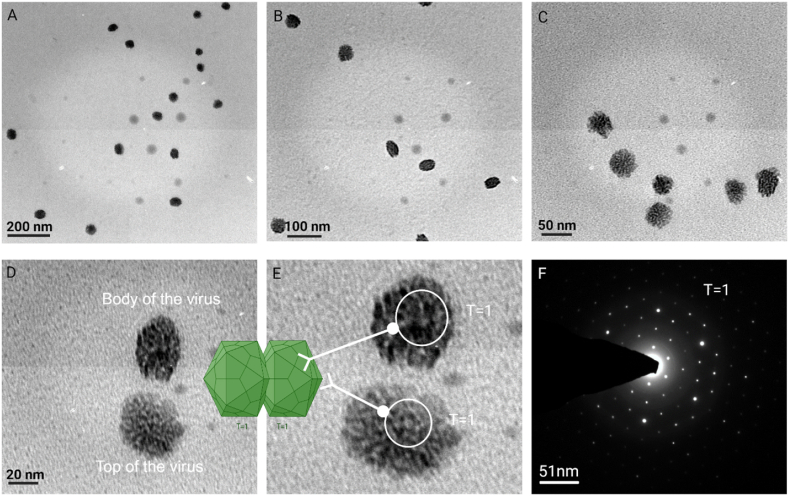
High-resolution transmission electron microscopy (HR-TEM) micrographs of crystalloid virus particles are presented at various magnifications, accompanied by bar scales for size reference. The images are as follows: A) 200 nm, B) 100 nm, C) 50 nm, D) 20 nm, E) a magnified image of virus particles, highlighting the clear aperture of the pentameric protein structure, and F) a Selected Area Electron Diffraction (SAED) pattern of a single virus particle, corresponding to the top particle in Fig. 1E. The graphical representation of geminivirus particle retrieved from the website https://viralzone.expasy.org/111.

For TEM imaging, the crystal was dissolved in buffer and a 1 μL drop of sample was loaded onto a carbon-coated copper grid (EM grids/Formvar/300 mesh/Sigma-Aldrich) positioned atop Whatman no.1 filter paper to eliminate any moisture. No staining or chemical fixation was employed for visualizing virus particles in the TEM. After allowing the samples to dry at 40 °C for 30 min, the grid was placed directly under the sample holder and analyzed in a TEM high-resolution transmission electron microscopy (HRTEM) (FEI, TechnaiG2, 30S-TWIN D905, USA). The HRTEM operated at an accelerating voltage of 250 KV, with a path length of −250mm (electron gun to specimen). To capture virus particle micrographs, different magnification ranges were used on the coated samples, resulting in five micrographs (Fig. 1 A-D).

Following image configuration, we conducted SAED on these images using a single virus particle to validate the crystalline nature of the virus particles. SAED is a widely employed technique in crystallography and materials science for analyzing the structural characteristics of nanoscale particles and materials [15]. It offers valuable insights into their crystalline structure and orientation [16]. This method entails directing a focused electron beam onto a specific region of the sample, resulting in electron diffraction patterns that unveil details about the arrangement of atoms or molecules within the material. In the context of virus particles, SAED can be an alternative for determining their crystal structure.

The absence of staining on the grid allowed for clear visualization of the virus particles, presenting them close to their natural state. Remarkably, the images also revealed the pentameric structure of the capsomer, a feature not previously observed in any of the literature (Fig. 1E) [[17], [18], [19], [20], [21]]. Moreover, many virus particles displayed their top and side portions within a single image frame, resembling the exact twin icosahedral shape of the Begomovirus [8,9]. The image quality was of a high standard, without cross-contamination, owing to the use of purified virus particles. The SAED pattern confirmed the crystal nature of the virus and further displayed the T = 1 symmetrical pattern (Fig. 1F).

Unlike other reports that relied on reconstruction software with undistinguishable cryo-TEM images, our study successfully visualized 2D and 3D kinds of images of viruses using standard TEM. The high image quality of viruses was achieved through the crystallization process, introducing a new avenue for virus structure analysis through traditional electron microscopy and overcoming challenges in specimen preparation.

#### 2.1.1 TEM-SAED analysis

**Fig. 2:**
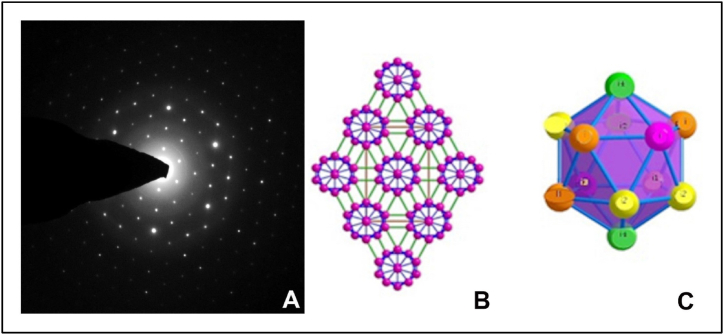
A. TEM-SAED pattern observations from a single crystalized virus particle, B & C the CrysTBox pattern analysis.

The icosahedral single crystal TEM SAED pattern analysis utilized the CrysTBox software, focusing on the Ti HCP (Titanium Hexagonal Close-Packed) structure mode. This analysis revealed two-dimensional icosahedral networks, which are pertinent to the structures of many icosahedral crystals. These networks encompass typical crystallographic planes, including (100), (010), (001), (110), (101), and (111) planes. The crystal structure of a novel phase was depicted as orthorhombic with the space group Pmna (Fig. 2 A). This structure comprises 24 atoms categorized into four non-equivalent atomic sites referred to as i1, i2, i3, and i4. These findings relate to the symmetry of spherical viruses, with icosahedra containing 60 subunits. Viruses are composed of multiple icosahedra of 60 subunits, forming slightly distorted icosahedral protein clusters. Watson and Crick proposed that cubic symmetry leads to isometric particles with an icosahedral (5:3:2) structure, consisting of 60 identical subunits. In this case, protein cluster units consist of 4i1 atoms, 4i2 atoms, 2i3 atoms, and 2i4 atoms. The center of Protein24 icosahedra projected along the [010] direction can overlap to form a rhombic packing (Fig. 2 B & C). In the T = 3 fold, there are four inequivalent atoms divided into two types: i2 and i4 atoms with five intra-icosahedral protein-protein bonds and one single inter-icosahedral i-i bond, while i1 and i3 atoms possess five intra-icosahedral i-i bonds and two double inter-icosahedral protein-protein bonds. It is widely accepted that inter-icosahedral bonding is characterized by covalent bonds and is stronger than intra-icosahedral bonding, as reported by Longuet-Higgins [22].

Considering the previous discussion, certain contradictions have emerged when comparing fundamental bonding concepts to experimental findings derived from HR-TEM SAED patterns. To reconcile these disparities, specialized geometric effects have been introduced, shedding light on the elastic responses exhibited by viral capsids. Notably, the analysis in Table 1 reveals the presence of varying bond strengths within icosahedral structures. The shortest bonds, represented by the inter-icosahedral i2-i2 bonds with a length of 0.165 Å, suggest their superior strength compared to the intra-icosahedral i-i bonds [23,24]. Conversely, weaker inter-icosahedral bonds, exemplified by i1-i1 (0.192 Å) and i1-i3 (0.204 Å) interactions, are observed. Furthermore, the examination uncovers additional inter-icosahedral interactions involving i1-protein, i2-amino acids, and i3-lipids, illustrating non-covalent bonding dynamics [25,26].

**Table 1:** Bonding analysis in icosahedral structure.

Pmna (no.532)	(a) 4.84		(b) 5.33		(c) 7.52		(V) 194.06	(r) 2.22
atom		position		x		y		z
i1		8i		0.699		0.3584		0.9343
i2		8i		0.8236		0.3400		0.1512
i3		4h		0.5000		0.6347		0.3040
i4		4h		0.500		0.8410		0.4915

## 3 Conclusion

This study represents a significant step forward in the field of virus structure analysis. By employing standard TEM without the need for toxic staining procedures, we have achieved remarkable high-resolution 2D and 3D images of Geminivirus particles. Our findings have unveiled some intricate structural properties of one of the most economically important groups of viruses worldwide, with unprecedented clarity. This was made possible by the simple crystallization method developed herein. The SAED pattern has also provided compelling evidence of the virus's natural icosahedral crystal arrangement, shedding new light on its architecture. Notably, this approach has allowed us to observe the pentameric structure of the capsomer, a feature previously undocumented in the literature for Geminiviruses. This innovative stain-free method eliminates the challenges associated with traditional electron microscopy techniques, such as cross-contamination during sample preparation and limitations in staining procedures. It opens up exciting possibilities for advancing virus research, diagnosis, and the understanding of viral diseases. In addition to its immediate impact on virus structure analysis, this breakthrough introduces a promising avenue for future investigations into any virus-related research, as well as applications in disease control and prevention. We hope that our work will inspire further exploration in the field and contribute to the development of effective strategies for combating viral diseases.

## Conflict of interest disclosure

The authors affirm that there are no competing interests to declare.

## Funding acknowledgment

This research was partially funded by the 10.13039/100000199USDA 10.13039/100005825National Institute of Food and Agriculture (10.13039/100005825NIFA) - 10.13039/100012125ARF Nano grant# GRANT13373733.

## Additional information

No additional information is available for this paper.

## CRediT authorship contribution statement

**Raja muthuramalingam Thangavelu:** Writing – review & editing, Writing – original draft, Formal analysis, Data curation, Conceptualization. **Washington Luis da Silva:** Writing – review & editing, Funding acquisition.

## Declaration of competing interest

The authors declare the following financial interests/personal relationships which may be considered as potential competing interests: Washington Luis Da Silva reports administrative support, article publishing charges, equipment, drugs, or supplies, statistical analysis, travel, and writing assistance were provided by Connecticut Agricultural Experiment Station. Raja muthuamalingam Thangavelu reports a relationship with Connecticut Agricultural Experiment Station that includes: employment. If there are other authors, they declare that they have no known competing financial interests or personal relationships that could have appeared to influence the work reported in this paper.
